# Fourth International Biannual Evolution and Cancer Conference (Resistance, resilience, and robustness: Can we target cancer's evolutionary and ecological nature?) Meeting report. Tempe, AZ, USA. December 7–10, 2017

**DOI:** 10.1111/eva.12616

**Published:** 2018-05-28

**Authors:** Benjamin Roche, Beata Ujvari, Frédéric Thomas

**Affiliations:** ^1^ Centre for Ecological and Evolutionary Research on Cancer Montpellier France; ^2^ UMI IRD/UPMC 209 UMMISCO Paris France; ^3^ Departamento de Etología Fauna Silvestre y Animales de Laboratorio Facultad de Medicina Veterinaria y Zootecnia Universidad Nacional Autónoma de México Delegación Coyoacán Ciudad de México CP México; ^4^ School of Life and Environmental Sciences Centre of Integrative Ecology Deakin University Geelong VIC Australia; ^5^ School of Natural Sciences University of Tasmania Hobart TAS Australia; ^6^ UMR CNRS/IRD/UM5290 MIVEGEC Montpellier France

## 1 INTRODUCTION

Many biological systems are resilient to shock and have the ability to return to a previous state following a disturbance. In the case of cancer, this resilience may jeopardize our understanding of tumorous cell proliferation and presents many clinical problems, including therapeutic resistance. Indeed, during progression and treatment, cancer has the capacity to exhibit resistance, resilience, and robustness, making its dynamics very challenging to forecast. Furthermore, organisms have evolved defenses that increase the robustness to mutations and other perturbations that can increase cancer susceptibility. Considering cancer and defense mechanisms to control oncogenesis through the lens of resilience and resistance can help identify challenges and opportunities in cancer therapy as well as expand the horizons for novel cancer prevention approaches.

The fourth biannual international Evolution and Cancer Conference of the International Society for Evolution, Ecology and Cancer (ISEEC), which had the theme “Resistance, Resilience and Robustness,” was held between December 7 and 10, 2017, in Tempe (AZ, USA). The biannual meeting aimed to bring together clinicians, theoreticians, and evolutionary scientists from all over the world to present the latest research developments in the field. Around 80 people attended the 2017 conference. Below we provide a report on the meeting, briefly summarize the plenary talks, and discuss the proceedings of the parallel sessions.

## 2 SUMMARY OF PRESENTATIONS

The meeting began on December 7 with a keynote address by Dr. Paul Turner (Yale University, CT, USA) on the evolutionary robustness of oncolytic RNA viruses. The first session, chaired by Dr. Carlo Maley (Arizona State University, AZ, USA), focused on the general theme “Evolution and Cancer.” The first speaker, Dr. Alexander Anderson from the Moffitt Cancer Center (Miami, FL, USA), talked about the evolution of cancer metaphenotypes. Using a hybrid multiscale mathematical model of tumor growth in vascularized tissue, the study showed that tumors develop heterogeneous spatiotemporal structures called metaphenotypes that collectively have an evolutionary advantage in the tumor. By categorizing each therapy response as a function of the initial tumor metaphenotype, drug sequences that promote a synergistic response can be identified.

Carlo Maley then discussed resistance management for cancer, especially drawing on knowledge from pest management that has led to three heuristic achievements in resistance management that could also be related to oncology. Maley explained that the overall aim is to transform cancer from a deadly disease into one that we can live with. This can be summarized as limiting the use of each mode of action (MoA) to the lowest practical level, diversifying the use of MoAs as much as possible, limiting each MoA to no more than two nonconsecutive uses, and partitioning MoAs in space or time so as to segregate their use as much as practically possible.

Dr. James DeGregori (University of Colorado, CO, USA) presented his research on the coevolution of somatic maintenance programs and mutation rates. Through stochastic modeling, he showed that the evolution of extended lifespans dramatically alters selection acting on germline mutation rates, significantly impacting on the ability to evolve while limiting somatic risks in populations of large animals. This may have been critical in enabling the evolution of large multicellular animals. In parallel, a new method of mutation detection allowing the observation of unselected mutations in normal tissues has shed new light on how somatic maintenance programs influence mutation rate tolerance (limiting tumor evolution) by impacting germline mutation rates and the variability of mutation rates in populations.

The work of Dr. Athena Aktipis (Arizona State University, AZ, USA) focuses on understanding how multicellular bodies “decide” if a cell poses a cancer threat. By developing a model relying on the cheater detection principle (benefits/costs of a false alarm, detecting cellular cheating where it is not happening), it becomes possible to predict how body size and longevity will influence selection on the information‐processing components of cancer suppression systems. Therefore, by applying cheater detection and signal detection theories to the problem of cancer suppression, we can better understand the function of complex gene regulatory networks that protect multicellular bodies from cancer and how they interact with other cancer suppression mechanisms such as immune surveillance.

Then, Dr. Aurora Nedelcu (University of New Brunswick, NB, Canada) presented her work exploring the role of selection in shaping cancer's evolutionary potential and resilience. After the application of several selective pressures on a cancer line that expresses adherent and nonadherent cells (to mimic cells in a solid tumor or circulating metastatic cells, respectively), the cells successfully evolved into five distinct cell lines that differ from the ancestral line in several traits related to fitness. Interestingly, although imposing a specific selective regime resulted in traits favoring adaptation to that environment, additional traits were also coselected. These traits (by‐products of selection) can either reduce or increase the fitness of the evolved line (relative to the ancestral line), depending on the environment.

Dr. Noemi Andor (Stanford University, CA, USA) presented her work on the identity of surviving and extinct clones in a longitudinal study of the DNA damage therapy response in gliomas. Overall, she showed that more than half of the clones detected among all patients were found across multiple biopsies of the same patient. Moreover, mutation profiles and clonal compositions from proximal biopsies were more similar to each other than those from distant biopsies. The study revealed a higher growth rate among clones with more amplifications but only among patients who had received DNA damage therapy.

This first session closed with the keynote talk was given by Dr. Christina Curtis on the way to quantify the evolutionary dynamics of therapeutic resistance and metastasis.

The second day of the meeting opened with a discussion panel that was chaired by Carlo Maley and entitled the “Future of Evolution, Ecology and Cancer.” The panel included Dr. Anna Barker (former Deputy Director of NCI), Dr. Alex Sekulic (Mayo AZ Cancer Center Director), and Dr. Dan Gallahan (Deputy Director of the Division of Cancer Biology at NCI). This was followed by a plenary session given by Dr. Deborah Gordon (Stanford University) on the ecology of collective behavior.

The first session of the day focused on ecosystem robustness and resilience. The first speaker, Dr. Frédéric Thomas (Centre for Ecological and Evolutionary Research on Cancer, CNRS, Montpellier, France), talked about the concept of oncobiota as an underappreciated component of animal evolutionary ecology. Indeed, given that malignant cells are omnipresent in the body of multicellular organisms, as are microbiota and parasites, they too may be involved in reciprocal interactions with the host phenotype. Therefore, malignant cells may also be involved in reciprocal interactions with microbiota and parasites, thus setting the scene for fascinating—yet complex—tripartite interactions; this appears to be a promising avenue to investigate.

The next talk, given by Dr. Beata Ujvari (Deakin University, Australia), was on adaptive evolution in the face of a transmissible cancer. While cancer is widespread in the animal kingdom, its impact on life history traits and strategies have rarely been documented. One exception is the devil facial tumor disease (DFTD), a transmissible cancer afflicting Tasmanian devils (*Sarcophilus harrisii*), where the phenotypic and genetic evolution of Tasmanian devils suffering from DFTD has been documented. This study shows that, akin to parasites, cancer can directly and indirectly affect devil life history traits and trigger host evolutionary responses.

Dr. Michael J. Metzger (Columbia University, NY, USA) next presented a study on the discovery a new kind of contagious cancer (leukemia‐like disease) in the soft‐shell clam (*Mya arenaria*), the Pacific blue mussel (*Mytilus trossulus*), the cockle (*Cerastoderma edule*), and the carpet shell clam (*Polytitapes aureus*). Transmission within each of these species is due to the independent horizontal spread of a clonal cancer lineage. However, while the cancer lineages in soft‐shell clams, mussels, and cockles are each derived from their respective host species, the cancer cells in *P. aureus* are derived from *Venerupis corrugata*, a different species that lives in the same geographic area but which itself is not known to be highly susceptible to disseminated neoplasia. These findings show that transmission of cancer in the marine environment is common in multiple species, that it has originated many times, and that both cross‐species transmission and species‐specific resistance occur.

Dr. Chandler Gatenbee (Moffitt Cancer Center, FL, USA) followed with a talk on the characterization of the immunogenic bottleneck. Based on a branching hybrid nonspatial cellular automaton, this study investigated whether the explosive antigenic diversity observed in colorectal cancer can be explained by either a “get lucky” strategy, where clones can have low enough antigenicity to avoid immune detection, or a “get smart” strategy, where clones can acquire active escape mechanisms. Only the “get smart” model is able to recapitulate the observed patterns of antigen burden and change in immune composition, suggesting that an active immune escape mechanism is required for carcinogenesis and implying that the immune system is the first treatment tumors must evolve resistance to.

The parallel session dealt with cancer evolutionary genomics. The first talk by Dr. Diego Mallo (Arizona State University, AZ, USA) presented the PISCA method, which is a new phylogenetic method for the reconstruction of somatic evolution using somatic chromosomal alteration data. This method, implemented as a plugin in the BEAST phylogeny software, is used to reconstruct the evolution of homogeneous somatic samples (i.e., single cells, single crypts, or deconvoluted clones) using somatic chromosomal alteration data. This method has been used to estimate the acquisition rate of somatic chromosomal alterations in Barrett's esophagus (BE) and its change through time. It has shown that the previously observed slow rate of evolution in this premalignant tissue is due to a low acquisition rate at the crypt level, explaining the low rate of progression from BE to esophageal adenocarcinoma by suggesting that clones with increased mutation rates appear to facilitate this transition.

The second talk, by Dr. Vincent Cannataro (Yale University, CT, USA), was entitled “The likelihood of heterogeneity or additional mutation in KRAS or associated oncogenes to compromise targeting of oncogenic KRAS G12C.” Because mutations in RAS genes are associated with approximately 20% of all human cancers, new targeted therapies inhibiting the KRAS G12C variant are very promising. Nevertheless, existing intratumor heterogeneity or de novo mutation can lead to resistance against these treatments. After having performed deep sequencing of 27 KRAS G12C‐positive lung tumors to determine the prevalence of other oncogenic mutations within KRAS or within commonly mutated downstream genes that could confer resistance at the time of treatment, patient‐derived xenografts were examined to assess the potential for novel KRAS mutations to arise during subsequent tumor evolution. No evidence of heterogeneity that could compromise the KRAS G12C‐targeted therapy within sequenced lung tumors or processed xenografts was found. These findings suggest that resistance of KRAS G12C‐positive tumors to targeted therapy is unlikely to be present at the time of treatment and, among the de novo mutations likely to confer resistance, mutations in BRAF (a currently available gene with targeted inhibitors) result in subclones with the highest fitness advantage.

The next talk was given by Dr. Luca Ermini (The Institute of Cancer Research, London, UK) on the evolutionary selection of cancer‐risk alleles. Analyzing genomes from five different Caucasian populations available in the 1,000‐genome database and using standardized methods to scan for genomic signatures of selection in gene loci associated with cancer risk, the aim of this study was to understand why cancer‐risk alleles are so frequent. While no (or neutral) selection was found for most alleles analyzed, a signal for positive selection was found in some variants associated with breast or prostate cancer in all populations analyzed and some population‐specific positive selection was found for some alleles associated with breast cancer. These results highlight new inroads into understanding the biological processes and evolutionary forces shaping cancer risk in humans.

The last talk of this session was given by Dr. Jeffrey Townsend (Yale University, CT, USA) on ways to quantify the intensity of natural selection on somatic mutations in cancer. Some high profile mutations have lower effect sizes than others whose *p* values are less significant but that exhibit a high effect size. Examination of the effect size conveys potential new targets for small populations, but also indicates that some high profile somatic nucleotide mutations (e.g., mutations in P53, even PIK3CA) have lower effect sizes than might be expected and may not have a successful therapeutic potential. Thus, a serious problem with using *p* values or mutation prevalence for ranking genes or mutations emerges from the same source that obviates use of genic mutation prevalence: the effect of mutation rate. Understanding the development of cancer as an evolutionary process permits the adaptation of classical evolutionary theory to use estimates of mutation rate to quantify selection intensity of mutation—cancer effect sizes. These effect sizes are the subject of analyses attempting to quantify the relative importance of mutations to tumorigenesis, cancer progression, and therapeutic resistance.

Following these parallel sessions, the poster flash talks took place prior to a plenary communication by Dr. Sunetra Gupta (Oxford University's department of zoology) entitled “Evolution and maintenance of pathogen population structure under immune selection.”

During the afternoon, the first session dealt with the evolution of therapeutic resistance. The first talk, given by Dr. Jill Gallaher (Moffitt Cancer Center, Tampa, FL, USA), concentrated on the possible exploitation of space and trade‐offs in drug scheduling to propose new cancer therapy. Assuming that evolutionary interactions may be crucial to identifying strategies to delay or prevent proliferation of the resistant population using conventional therapies, an agent‐based framework has been developed to model competition among sensitive and resistant populations during therapy in a spatially competitive resource‐limited tumor microenvironment. It has been found that tumors consisting only of sensitive cells can be cured with continuous treatment, which is permanent treatment at the maximum tolerated dose, but the presence of resistant cells will lead to eventual recurrence. In this case, strategies emphasizing continuous dose modulation or relying on treatment vacations can control tumor expansion.

The second talk was given by Dr. John Nagy (Arizona State University, AZ, USA) and was related to the development of a model of natural selection that could predict treatment resistance in prostate cancer. Formulated and parameterized with a sample of 25 patients treated with intermittent androgen‐ablation therapy, this adaptive dynamics model of androgen‐ablation therapy was then used to predict PSA dynamics in an independent set of 30 patients from the same clinical study. While predictions were usually reasonably accurate for one cycle, and for some patients up to four cycles, this model had some significant exceptions that can be explained by resistance arising from different mechanisms. Therefore, this modeling approach may provide a noninvasive method to identify emerging resistance mechanisms in nascent hormone‐refractory tumors and to plan treatment to delay development of castration resistance.

Dr. Daniel Nichol (Institute of Cancer Research, London, UK) then showed how stochasticity in the genotype–phenotype map can have implications for the robustness and persistence of the “bet‐hedging” strategy in cancer cell populations. Drug tolerance mechanisms have been observed without apparent genetic drivers, suggesting that bet‐hedging may play a role in driving resistance. Through a simple model involving a molecular switch, it was possible to demonstrate that bet‐hedging is resistant to loss from mutations in both the expression of genes and their interactions, suggesting that single‐gene knockouts may be insufficient to elucidate the drivers of bet‐hedging. The implications for therapy have been investigated, highlighting that the successful attempts to “steer” the evolution of bet‐hedging through drug holidays will be dependent on the G–P mapping.

The parallel session was on the evolution of cancer suppression mechanisms and organism robustness. The first speaker, Dr. Marc Tollis (Arizona State University, Tempe, AZ, USA), discussed a molecular evolutionary approach to understanding cancer suppression, and especially Peto's paradox. While large species should face a higher lifetime risk of cancer due to the greater probability of oncogenic mutations occurring during somatic evolution, zoo necropsy data reveal that elephants have a ~5% probability of death from cancer compared to 11%–25% for humans. This study showed that elephant genomes harbor up to 40 alleles of the tumor suppressor gene TP53. Moreover, functional assays demonstrate that TP53 redundancy in elephants is related to an increased apoptotic response to DNA damage in elephant cells when compared to human cells. Across >50 mammalian genomes, multiple tumor suppressor gene copy‐number expansions have been found to co‐occur with the evolution of large body size or longevity in elephants, bats, horses, and rhinos, suggesting that convergent evolution toward large bodies and long lifespans was accompanied by adaptive checks on neoplastic progression. These results show that nature won over cancer numerous times and that the comparative genomic signatures of adaptation in mammals can help expand “nature's toolkit” for cancer prevention and potentially improve clinical outcomes for humans.

The subject of the second talk, given by Dr. Benjamin Roche (Centre for Ecological and Evolutionary Research on Cancer, Montpellier, France), was how nononcogenic infectious agents modulate cancer development through the alteration of immune responses. By inducing immunosuppression (from a cancer cell perspective), nononcogenic infections could be indirectly detrimental to the host by permitting cancer cell accumulation. Using experimental infections in a larval *Drosophila* brain tumor model analyzed with a combination of image analysis, transcriptomic study of immune gene expression, and sophisticated statistical modeling, it has been possible to demonstrate that larvae infected with the bacterium *Pectobacterium carotovorum carotovorum* showed a smaller tumor size compared to control and fungi‐infected larvae. This reduction was associated with an increased expression of the associated immune pathways, showing an indirect interaction between nononcogenic infectious agents and cancer development through altered immune responses.

The last talk of this session was delivered by Dr. Amy Boddy (University of California, Santa Barbara, CA, USA), who presented a large‐scale evaluation of neoplasia occurrence and life history traits in vertebrates. Through a compilation of necropsy reports from pathology datasets and an estimate of cancer prevalence across vertebrates, this study showed that—consistent with previous estimates—the occurrence of neoplasia is higher in mammals (25%) than in reptiles (14%), birds (10%), or amphibians (4%). This current dataset has body masses ranging from 0.004 kg in the green anole to 4,540 kg in the African elephant and maximum lifespans ranging from two years in the carmine bee‐eater to 80 years in the African elephant. The domestic ferret had the highest incidence of reported of neoplasm (70% of individuals, *n* = 4,000), and alligators had the least (1% of individuals, *n* = 290). In support of Peto's Paradox, a negative relationship between cancer incidence and body mass and lifespan was observed.

The day ended with a public lecture by Dr. Elizabeth Murchison (Cambridge University, Cambridge, UK) on transmissible cancers in dogs and Tasmanian devils.

Saturday, December 9, began with a plenary communication by Grazyna Jasienska (Jagiellonian University Medical College, Krakow, Poland) entitled “The evolution of female reproduction and breast cancer: it was never about the 3 Rs.”

Following this, the first session on evolution of therapeutic resistance began with a talk by Dr. Ahmet Acar (Institute of Cancer Research, London, UK) on quantitative measurements of treatment resistance in nonsmall‐cell cancer. This study involved the development of a model system, initially in lung cancer cell lines, that enables the measurement of evolutionary dynamics in vitro using semirandom DNA barcode sequences introduced into cell lines via lentiviral transduction. This method includes a high‐throughput drug screen of resistant clones to discover acquired sensitivities to new drugs combined with mathematical modeling to determine fitness landscapes and predict which sequences of small molecule inhibitors may be used for sensitizing a cancer cell population to the next inhibitor. This method provides a framework to test and choose treatment options before they are considered for in vivo and clinical applications.

The second talk, by Dr. Rob Noble (ETH Zurich, Switzerland), focused on how spatial competition constrains resistance to targeted cancer therapy. Cancer cells that are resistant to a pharmacological cyclin‐dependent kinase inhibitor (CDKi) are generated; these cells have reduced proliferative fitness and stably rewired cell cycle control pathways. Mathematical modeling indicates that the tumor's spatial structure amplifies the fitness penalty of resistant cells and identifies their relative fitness as a critical determinant of the clinical benefit of adaptive therapy.

Dr. Jeffrey Chuang (The Jackson Laboratory for Genomic Medicine, Farmington, CT, USA) presented a study on the evolutionary dynamics of response to chemotherapies in breast cancer xenografts. This study showed how it is possible to finely resolve evolution in response to multiple chemotherapies by sequencing post‐treatment residuals from patient‐derived xenografts (PDXs) grown from two triple‐negative breast cancer patients combined with exome‐sequencing and 1,633 droplet digital PCR (ddPCR) measurements for mutation and CNV quantitation. Using assays of 86 xenografts and 45 derived cell cultures, it was possible to distinguish selection from measurement uncertainty, intraclonal diversity, and spatial drift, with improvements over inferences from exome‐sequencing data. Common modes of evolution within these tumors have been observed, including population bottlenecks, spatial diffusion, and stable coexistence between distinct subpopulations. Notably, it has been possible to show that a major pre‐existing subclone exhibited higher cisplatin sensitivity but was favored when treatment was suspended, indicating an ecology susceptible to retreatment by adaptive therapy. This demonstrates the importance of intratumoral dynamics in guiding treatment strategy.

The topic of the next talk, given by Dr. Benjamin Werner (The Institute of Cancer Research, London, UK), was about forecasting resistance evolution in cancer from liquid biopsies. After discussing some approaches on how this heterogeneity might be better classified from multiregion sequencing data and how this might improve the selection for potential targets of treatment, this study has shown how sequential sampling of circulating tumor DNA (ctDNA) in patients during treatment can be used to forecast the evolution of treatment resistance. Interestingly, a combination of sequential sampling and evolutionary modeling does not only detect resistance but also allows quantifying some properties of the evolutionary process.

Dr. Nara Yoon (Cleveland Clinic Foundation, OH, USA) followed with a talk on optimal chemotherapy scheduling based on a pair of collaterally sensitive drugs. To avoid drug resistance, researchers have proposed sequential drug therapies so that the resistance developed by a previous drug can be relieved by the next one, a concept called collateral sensitivity. In this study, dynamic models were developed and revealed that the optimal treatment strategy consists of two stages: (Stage 1) the initial stage in which a chosen “better” drug is utilized until a specific time point, *T*; and then (Stage 2) a combination of the two drugs with a relative intensity (*f*) for Drug A and (1‐*f*) for Drug B. Importantly, the initial period during which the first drug is administered, *T*, has to be shorter than the period in which it remains effective, contrary to clinical intuition.

The next talk was given by Dr. Andriy Marusyk (Moffitt Cancer Center, Tampa, FL, USA), who has shown that acquired resistance to targeted therapies evolves through gradual, therapy‐directed trajectories. The lack of evolutionary dynamics and trajectories or resistance acquisition remain unexplored, partly because the dominance of an assumption that resistance emerges as the result of a binary (epi) mutational switch, which suggests that relapse of the disease could be reduced to a selective expansion of resistant clones and little can be done clinically to interfere. This was challenged by investigating how the evolution of resistance toward multiple clinically relevant ALK‐TKI emerges using in vitro models of EML4‐ALK+ lung cancers. Exposure of EML4‐ALK+ cell lines to different clinically relevant ALK‐TKI leads to the rapid and reproducible development of resistance. Resistance evolves gradually, originating from weakly resistant precursors and culminating in the (near) complete loss of growth inhibition. Even though the end products of this evolution converge to pan‐ALK‐TKI resistance, different ALK‐TKI selects for predictably distinct molecular adaptations that are associated with distinct cross‐sensitivities. These observations suggest that explicit consideration of evolutionary dynamics could lead to the development of novel approaches to block or delay evolution of resistance.

The parallel session concerned cell viability in the face of genomic alterations, and the first talk was given by Dr. Violet Kovacheva (Institute of Cancer Research, London, UK). This talk focused on the application of an automated image analysis system that analyzes the morphology and texture of all epithelial cells at single‐cell resolution within ductal carcinoma in situ (DCIS) of the breast to identify the morphological variability present within the tissue sample, allowing the DCIS region to be classified as high or low nuclear grade. This method has an accuracy of 85.4% when compared with at least one of two pathologists’ grades (in comparison, the two pathologists agreed on the grade in 73.8% of independently evaluated cases), demonstrating that automated histology image analysis generates clinically relevant grading for DCIS.

The following talk, by Dr. Enrico Borriello (Moffitt Cancer Center, Tampa, FL, USA), dealt with how network duplication reinforces phenotypes by increasing attractor basin sizes. The current prevailing explanation for whole‐genome duplication is that the extra copies of genes preserve function while allowing cells to explore possible adaptations. After modeling this hypothesis within the idealized framework of small arbitrary Boolean networks, and defining a phenotype as all network states sharing some specific activation pattern of a given subset of nodes once the network has converged into a dynamic attractor, it has been observed that network duplication affects the relative sizes of the basins of attractions, very often increasing the size of larger basins at the expense of smaller ones. This theoretical result was consistent with an analysis of a Boolean representation of the p53‐dependent DNA damage response network at the G1 checkpoint. This opens new opportunities for understanding the therapeutic response of cancer, which is often characterized by whole‐genome duplication followed by chromosome loss.

The next talk was given by Dr. Kelsey Temprine (Memorial Sloan Kettering Cancer Center, New York, NY, USA), with the topic being how the ability of melanoma to evolve is mediated by DNA polymerase kappa. While bacteria resistance can evolve via induction of the error‐prone DNA polymerase DinB during stress‐induced mutagenesis, it can be hypothesized that melanoma cells under drug stress could use similar mechanisms by upregulating DNA polymerase kappa (polκ), the vertebrate homolog of DinB. Using human melanoma cell lines and a zebrafish model of melanoma, polκ's mRNA, protein, and subcellular localization after MAPK inhibition were examined. As a result, treatment of human melanoma cell lines with MAPK inhibitors led to a significant increase in polκ mRNA levels and a dramatic protein shift from the cytoplasm to the nucleus. In the zebrafish model, constitutive overexpression of polκ accelerates melanoma formation and also yields atypical tumors. The mechanism behind this phenomenon is currently being investigated and may provide important insights into tumor evolution, providing new opportunities for reducing the chances of acquiring resistance.

Next, Dr. Peter J. O'Brien (Pfizer, San Diego, CA, USA) showed how a single gene modulates stressed cell resilience. Previously obscured by the persistent use of nonstandard nomenclature, stress‐sensitive telomere‐ and ribosome‐accessory proteins (stressTRAPs) constitute a novel family of eukaryotic stress‐resilience regulators. StressTRAPs are tightly regulated and differentially expressed in response to a variety of cell‐intrinsic and cell‐extrinsic homeostatic challenges, modulating cell fates via effects on chromatin dynamics, RNA metabolism, and protein translation. StressTRAP influences on tissue development and expansion, reproductive timing, metabolism, energy budgeting, and lifespan suggest that they help align resource consumption with organismal health and environmental conditions, and may explain SERBP1 disease associations, which is universally expressed in cancer. In addition, its expression correlates with tumor aggressiveness while its mutational intolerance may obscure links to other diseases. The remarkable functional conservation across multiple species described in this study suggests that these problems are tractable in lower eukaryotic model systems and encourages additional mechanistic studies.

The next talk, given by Dr. Henry Heng (Wayne State University, Detroit, MI, USA), presented work distinguishing how gene mutation mediated microcellular evolution from karyotype reorganization as well as mediated macrocellular evolution in cancer. Different concepts were examined in this study to illustrate how chromosomes drive cancer evolution: (i) The karyotype represents a new type of genomic coding. By determining the order of genes along and among chromosomes, the genome organizes the gene interaction map accordingly; (ii) different coding patterns contribute to different types of cancer evolution. Gene mutations are crucial for microcellular evolution while karyotype alterations are necessary for macrocellular evolution. Macro‐evolution is not simply an accumulation of micro‐evolutionary processes over time; (iii) cancer evolution is highly unpredictable during the punctuated phase, where genome chaos dominates; (iv) when gene mutations are insufficient for cell survival, genome reorganization will occur as a survival strategy; (v) the average profile of cancer cells cannot predict drug resistance due to outliers. Challenging these concepts with experimental data illustrates how cancer evolution offers a unique window to understanding evolutionary principles.

Finally, the last talk of this session was given by Dr. Kimberly J. Bussey (NantOmics, Phoenix, AZ, USA), who showed that a noninherited mutation is constrained by the genomic evolutionary history in nonintuitive ways. This study identified the noninherited (de novo) single nucleotide variants (SNVs) in 129 individuals using the methodology of somatic variant calling. Through different data treatments, it was observed that the SNVs filtered out had different evolutionary properties depending on the filter applied. SNVs that were filtered out at data quality control stages were enriched for regions of the genome that pose difficulties for unique alignment, such as segmental duplication and inversion regions (SDRs) and nonallelic homologous recombination (NAHR) substrates, but not LTRs. In contrast, data filtered out by allele frequency were enriched in LTRs and homologous synteny blocks and excluded from SDRs, NAHRs, and evolutionarily re‐used breakpoints. Additionally, SNVs filtered at the data quality steps were slightly enriched to be in clusters of variants while those filtered by allele frequency were excluded from clusters, suggesting that clustering is dominated by somatic/early germline events. In general, SNVs preferentially affected genes younger than 1,500 MY, but strong filtering tends to create a bias against recovering this pattern.

This session was followed by a plenary talk by Dr. Pablo Marquet (Pontificia Universidad Catolica de Chile, Santiago de Chile, Chile) on the relationships between diversity, transitions, and robustness in ecosystems. A first parallel session was then held on theoretical evolutionary biology of robustness in cancer; this began with a communication by Dr. David Basanta (Moffitt Cancer Center, Tampa, FL, USA), who used the definition of the bone ecosystem to understand how selection in prostate cancer can be linked to bone metastasis. This study argues that bone homeostasis is an important regulator of prostate cancer metastasis in the bone and that understanding the mechanisms of bone homeostasis is thus key if we want to understand and target the prostate cancer phenotypes that can disrupt it. To do so, a combination of experimental and mathematical models offers the best hope to tackle the complexity of this endeavor, and this seems to be a very promising research avenue.

The second talk, given by Dr. Weini Huang (Queen Mary University of London, London, UK), revealed the evolutionary mechanisms of spatial mixing of subclones in tumor by a mathematical model and colorectal tumor samples. This work relied on a stochastic spatial model of a mutant arising in a wild‐type tumor population to assess subclonal mixing patterns, which can show how this spatial information can reveal the underlying evolutionary dynamics. Monitoring the mutant frequency and the mixing score (Shannon's entropy) over time shows that an intermediate selection advantage for the mutant type will lead to the highest mixing among the wild type and the mutant, producing a hump‐shaped curve of mixing scores (visually assessed) over time after the mutant arises.

The next talk was given by Dr. Dominik Wodarz (University of California, Irvine, CA, USA) and dealt with the impact of evolutionary dynamics and treatment on feedback regulation in cancer. Through the development of mathematical models of feedback regulation in healthy tissue and cancer, the impact of such regulatory processes on the dynamics of tissue and cancer stem cells was investigated. The evolutionary dynamics of escape from negative feedback regulation was discussed. Moreover, the possibility that certain feedback loops characteristic of healthy tissue remain functional to a certain extent in tumors was investigated, and this was revealed by the observation of specific growth patterns in tumors. Finally, the speaker discussed the impact of such feedback loops in tumor tissue on cancer stem cell enrichment during treatment, which can lead to the development of stem cell‐based therapy resistance.

The other parallel session concentrated on cancer prevention such as resilience/robustness in the face of somatic challenges and began with a talk by Dr. Elena Svenson (Case Western Reserve University, Cleveland, OH, USA) on the quantification of the effects of advantageous, deleterious, and neutral passenger mutations on variant allele frequencies (VAF) architecture. Only driver mutations in the background of neutral evolution have been considered so far, even though this approach could lead to the possibility that modeled time of acquisition and selective advantages could be incorrectly estimated because it is hypothesized that many passenger mutations could be slightly deleterious. Through the development of a stochastic model of tumor evolution simulating neutral, beneficial, and deleterious mutations propagating through the population, this model provides a more general model for VAF architecture. It has therefore been possible to show how VAF distributions change based on differing strengths of deleterious and advantageous events, offering possible clarity in cases where it is difficult to tease out neutral evolution alone. These distributions are then fit, using approximate Bayesian computation, to whole‐exome data from The Cancer Genome Atlas, to determine whether the effects of deleterious passenger mutations could explain distributions seen in human tumors. These results provide a new way with which to understand the subtleties associated with the analysis of clonal population dynamics in bulk genomic sequencing data.

The next talk by Dr. Angelo Fortunato (Arizona State University, Tempe, AZ, USA) was on the development of novel model organisms in cancer research. Because some invertebrate phyla have no reports of cancer, these species must be particularly resilient to mutations or rely on highly effective molecular mechanisms of damage prevention, DNA repair, or tissue‐level cancer control that are worth investigating. This has been tested on three invertebrates for which there have been no reports of cancer: *Trichoplax adhaerens* (Placozoa), *Tethya wilhelma* (sponge), and *Macrostomum lignano* (flatworm). Dr. Fortunato observed that *T. adhaerens* have an elevated resistance (~160 Gy) to X‐rays, where cellular aggregates with a different morphology were observed after several weeks of high‐dose exposure (even though it is unsure that these were a form of cancer). *T. wilhelma*, which adapts well to being cultured in a laboratory setting, is even more resistant to X‐rays (~700 Gy). Finally, the flatworm *M. lignano* has an elevated regenerative ability conferred by its high percentage of stem cells (the highest recorded in an animal) and is much less resistant to X‐rays (~60 Gy).

This session ended with a talk by Dr. Pierre Martinez (Cancer Research Center of Lyon, Lyon, France) on the evolution of Barrett's esophagus (BE) through space and time at single‐crypt and whole‐biopsy levels. In this study, researchers noted copy‐number alterations (CNA) from SNP arrays in 6–11 biopsies over two time points in each of eight individuals with Barrett's esophagus, including four cancer progressors. Eight individual crypts and the remaining epithelium were assayed for each biopsy, yielding 358 valid samples. This allowed the characterization of genetic diversity at an unprecedented resolution and the reconstruction of corresponding phylogenies. In six patients, CNAs could be detected in all crypts and biopsies, suggesting lesions derived from a single ancestor. While crypts contained private mutations, mutational load and rates in crypts were similar to those in whole biopsies; thus, biopsies were adequate for evolutionary studies. Moreover, Dr. Martinez observed that “macrodiversity” between biopsies reflected the “microdiversity” between crypts of a biopsy, that genetic distances between crypts were unrelated to physical distances, and that rare clonal expansions indicated that BE lesions are mostly evolving neutrally. These results shed new light on the evolutionary dynamics underlying BE genetic evolution and reveal they are adequately described by biopsy‐level macroscopic heterogeneity.

These two parallel sessions were followed by a plenary talk by Dr. Jake Scott (Cleveland Clinic, Cleveland, OH, USA) on how we can learn and perturb the evolutionary mechanisms driving therapeutic resistance in cancer. The last day (Sunday, December 10) began with a plenary by Dr. Inigo Martincorena (Wellcome Sanger Institute, Cambridge, UK) on the somatic evolution in normal tissues, followed by another plenary by Dr. Bruce Tabashnik (University of Arizona, Tucson, AZ, USA) on what we can learn from insect resistance to transgenic crops. The conference ended with closing remarks from Carlo Maley and Athena Aktipis. This conference was an additional evidence that the field Evolution and Cancer is maturing and moving toward bringing genuine alternative and creative ways to understand and fight cancer.

## CONFLICT OF INTEREST

None declared.

